# Flexible optofluidic waveguide platform with multi-dimensional reconfigurability

**DOI:** 10.1038/srep33008

**Published:** 2016-09-06

**Authors:** Joshua W. Parks, Holger Schmidt

**Affiliations:** 1 School of Engineering, University of CA Santa Cruz, 1156 High Street, Santa Cruz, CA 95064 USA

## Abstract

Dynamic reconfiguration of photonic function is one of the hallmarks of optofluidics. A number of approaches have been taken to implement optical tunability in microfluidic devices. However, a device architecture that allows for simultaneous high-performance microfluidic fluid handling as well as dynamic optical tuning has not been demonstrated. Here, we introduce such a platform based on a combination of solid- and liquid-core polydimethylsiloxane (PDMS) waveguides that also provides fully functioning microvalve-based sample handling. A combination of these waveguides forms a liquid-core multimode interference waveguide that allows for multi-modal tuning of waveguide properties through core liquids and pressure/deformation. We also introduce a novel lifting-gate lightvalve that simultaneously acts as a fluidic microvalve and optical waveguide, enabling mechanically reconfigurable light and fluid paths and seamless incorporation of controlled particle analysis. These new functionalities are demonstrated by an optical switch with >45 dB extinction ratio and an actuatable particle trap for analysis of biological micro- and nanoparticles.

The integration of photonic functions with non-solid media has made tremendous progress in recent years^1^,^2^,^3^,^4^,^5^,^6^,^7^,^8^,^9^,^10^,^11^,^12^,^13^,^14^,^15^,^16^,^17^,^18^. Such optofluidic integration is particularly attractive for creating reconfigurable optical devices and for taking lab-on-chip based approaches for biological and chemical analysis to new levels. Photonic elements that can be altered by introducing different fluids or changing device dimensions with applied pressure include lasers^3^,^4^,^5^,^6^, spectral filters^7^,^8^,^9^,^10^, lenses^11^, interferometers^12^,^13^, and optical switches^14^,^15^,^16^,^17^. Lab-on-chip style optofluidic analysis, on the other hand, has led to the development of waveguide-based biomolecular analysis down to the observation of single nucleic acids and viruses^19^,^20^,^21^,^22^. Up to now, the most sensitive devices have predominantly taken a hybrid approach^23^,^24^ in which microfluidic sample handling is done in PDMS chips while ultrasensitive optical detection is implemented in semiconductor based solid-state waveguides.

Here, we introduce a new optofluidic platform that provides both multi-modal photonic reconfiguration and advanced fluidic sample handling in a single chip. On-chip photonic devices are based on a combination of solid-core and liquid-core PDMS waveguides as shown in Fig. 1a. The waveguides can be built with established multilayer soft lithography techniques and seamlessly connect with each other to form a wide variety of photonic layouts. Moreover, the layer structure is compatible with incorporation of fluidic microvalves to enable both optical tuning and fluid control in a single device. Specifically, the index-guiding solid-core waveguides are formed by controlling the PDMS precursor ratio^25^ in different layers to create a high-index waveguide core (for more details see the Methods section). Unlike previous implementations of PDMS waveguides, our cores are only ~7 × 8 μm in cross section (unless otherwise noted), providing excellent mode matching to single-mode fiber, thus allowing for advanced photonic devices such as interferometers that rely on careful control over one or a few waveguide modes. Liquid-core sections are either filled with high-index liquid to enable index-guiding or kept short to minimize optical losses in low-index leaky mode operation.

## Results

**Figure 1:**
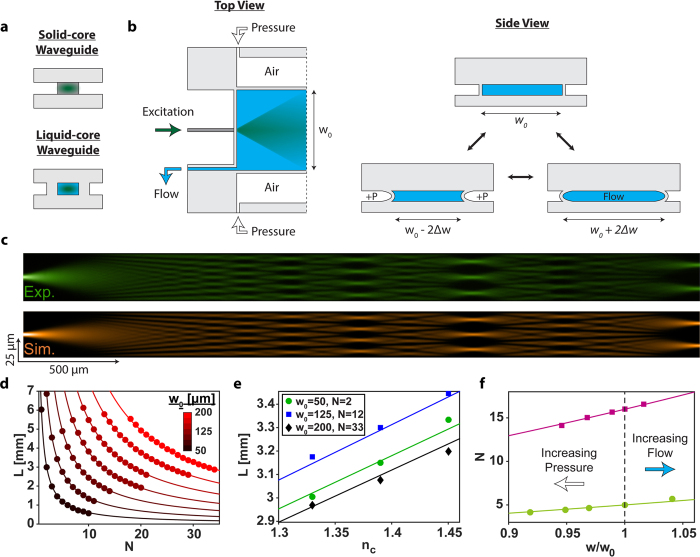
Dynamically tunable multispot optofluidic waveguide. (**a**) Schematic side-view of the solid- and liquid-core waveguides. (**b**) Liquid-core MMI waveguide system from top (left) and side (right) views. Fiber injected laser light propagates through the excitation solid-core waveguide and into the liquid-core waveguide with a static width of w_0_. Air pressure yields a decrease in liquid-core waveguide width (bottom left, side view) while liquid flow increases waveguide width (bottom right, side view). (**c**) Experimental (top, green) and simulated (bottom, orange) multispot waveguide pattern for a 50 μm wide liquid-core waveguide. (**d**) MMI length vs. spot number for 7 fabricated liquid-core waveguide widths, 50–200 μm, 25 μm increment. (**e**) Dynamic tuning of optofluidic waveguides by varying core refractive index, n_c_. (**f**) Width based dynamic tuning of optofluidic waveguides, w_0_ = 50 and 100 μm for green circles and magenta squares. Positive pneumatic pressure points increase to the left, at 20, 40, and 60 PSI, while the liquid flow rate is 1 mL/min. Note: theoretical predictions using eq. (1) are represented as solid curves/lines in (**d–f**).

In order to demonstrate the physical implementation of the PDMS waveguide platform and the ability to tune an optical device using both fluid control and pressure, we first consider a multi-mode interference (MMI) waveguide^26^. MMIs create length and wavelength dependent spot patterns upon propagation of multiple waveguide modes, and have recently been used to implement spectrally multiplexed detection of single viruses flowing through intersecting fluidic channels^21^. Our liquid-core optofluidic MMI is schematically shown in Fig. 1b and is designed for active tuning by varying both pressure and core fluid. A 5 μm wide and 7 μm tall solid-core waveguide (dark grey) is used as an input for the wide liquid-core MMI section (width *w*_*0*_, length *L*). The MMI is surrounded laterally by 50 μm wide air channels, which enable both optical waveguiding and tuning of the MMI width through pneumatic and fluidic pressure, as is illustrated in the right side of Fig. 1b.

The multimode interference leads to the formation of *N* images of the input mode for a given length, *L*, and pressure, *P*, according to


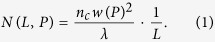


This pattern formation is visualized in Fig. 1c (top) for a static MMI (*P* = 0; *w*_*0*_ = 50 μm) filled with fluorescent dye in ethylene glycol (*n*_*c*_ = 1.45) and excited with λ = 532 nm laser light. Clean spot patterns are observed over a distance of several millimeters in excellent agreement with eq. (1) and finite difference method simulations shown in Fig. 1c (bottom). Liquid-core MMIs with widths between 50 and 200 μm (25 μm increment) were fabricated and characterized as presented in Fig. 1d. We were able to controllably vary the spot number from 1 to 34 images with device lengths less than 1 cm, all in excellent agreement with theory (lines). Such MMIs, therefore, provide a wide parameter space for multi-spot particle detection with high signal-to-noise ratio^21^,^27^.

Next, we turn to dynamic tuning of these optofluidic elements. The first mechanism is through replacement of guiding liquid, i.e the waveguide core refractive index, *n*_*c*_. Fig. 1e shows MMI tuning using different mixtures of ethylene glycol and water. Specifically, a sampling of waveguides (with various widths, *w*_*0*_, and spot numbers, *N*) were used to demonstrate the linear relationship between core refractive index, *n*_*c*_, and image length, *L*. Tuning of the spot number over a very wide range from 2 to 33 was realized, and excellent agreement between theoretical and experimental results was found.

Thin sidewalls made from a pliable material (PDMS) allow for controlling a microfluidic channel’s width through both inward and outward pressure^28^. Here, we use this principle for pressure-based dynamic tuning of the optofluidic MMI devices. Inward pneumatic pressure applied to the side channels causes a decrease in the MMI width, (Fig. 1b, right) and thus, a decreased spot number, *N*, at a given length, *L*. Conversely, positive fluidic pressure in the core increases both *w* and *N* as seen in Fig. 1b,f. Note that all data points in Fig. 1f are at a given length *L* that yields an integer spot number at zero applied pressure. The data closely matches theoretical expectations (lines in Fig. 1f). Furthermore, there is no notable decrease in fluorescence signal during sidewall deformation, indicating negligible effects on the optical loss of the waveguide.

**Figure 2:**
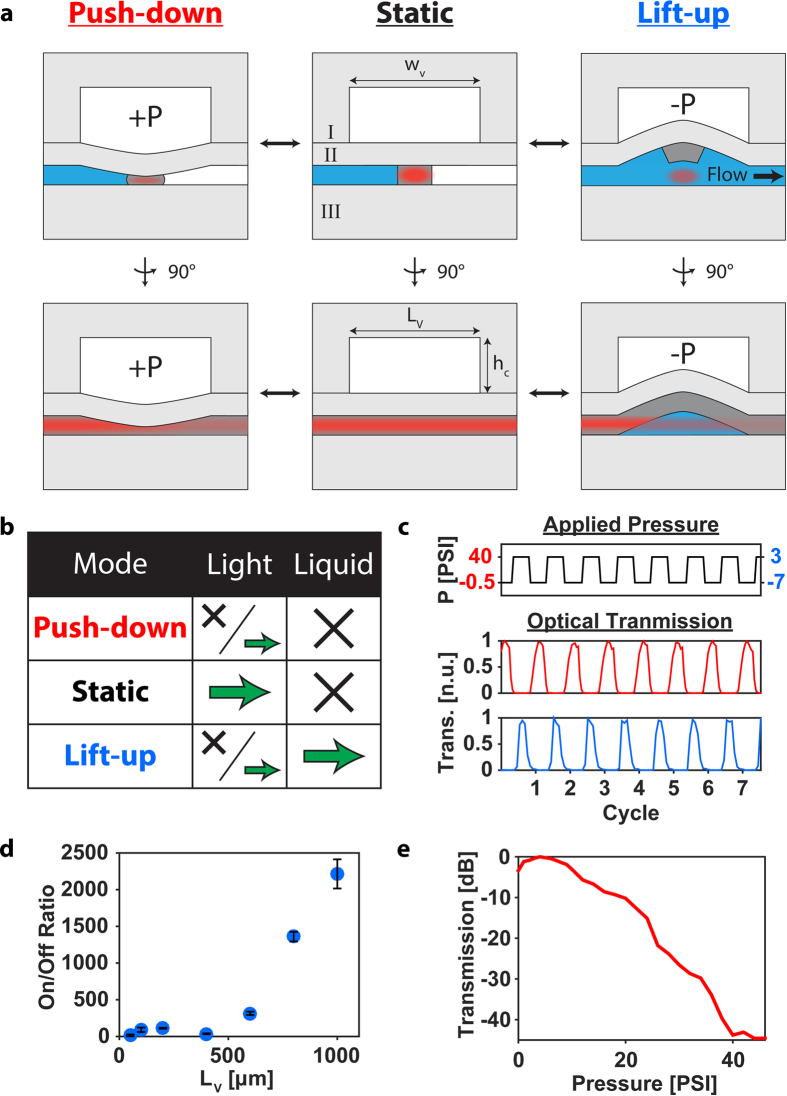
Lightvalve design, function, and operation. (**a**) Schematic representation of the lightvalve architecture. Top-center—as fabricated lightvalve composed of three functional layers: layer I is the control layer; layer II is composed of a high refractive index waveguide core (dark grey) and a low refractive index mechanical membrane (light grey); layer III is the substrate layer. Top-left—lightvalve operated in push-down mode. Top-right—lightvalve operated in lift-up mode. The bottom row of images are respective counterparts to the upper row, rotated 90° about a vertical axis. Importantly, the control layer defines the lightvalve’s width (w_v_) and length (L_v_) as well as the maximum deflection of layer II via its height, h_c_. (**b**) Lightvalve operation and respective effects on liquid and light flow. Note that light flow is tailorable/tunable in both lift-up and push-down modes (see parts **d,e**). (**c**) Optical switching of the lightvalve operated in lift-up (bottom, blue) and push-down (red, middle). The top subplot (black) is a schematic pressure trace designating the applied control pressure at any given time. The left-hand y-axis (red labels) denotes the pressure applied during push-down operation while the right-hand y-axis (blue labels) denotes the pressures applied in lift-up mode. (**d**) Optical switching efficiency of lightvalves operated in lift-up mode. The error-bars represent the standard deviation of 20 switching cycles. (**e**) Optical rejection of a lightvalve operated at various push-down pressures.

We now turn to introducing a new approach for a fully–optically and fluidically–reconfigurable optofluidic platform. At its heart is an actuatable microvalve that simultaneously acts as an optical waveguide and actively moderates fluid flow, dubbed here as a “lightvalve”. Our implementation is based on lifting-gate microvalves that have been used in microfluidic devices for complex bioassays^29^,^30^. Figure 2a shows the schematic design of the lightvalve, with the middle images showing its static architecture in cross-section and side view. It is composed of three PDMS layers, a control layer (I), a waveguide valve layer (II), and a substrate (III). The control layer, I, is designed to allow for both push-down (positive pressure, Fig. 2a, left) and lift-up (negative pressure, Fig. 2a, right) operation. By varying the pressure, different combinations of photonic and fluidic functions of the lightvalve can be implemented as shown in the table of Fig. 2b. Without pressure, light is guided across the fluid valve (green arrow) while liquid flow is blocked (cross). Alternatively, positive pressure also blocks liquid flow while enabling dynamic tuning of optical transmission by varying the pressure. Finally, negative pressure results in fluid flow with tailorable optical rejection. The lightvalve can be constructed with established lifting gate valve fabrication processes, plus the addition of the waveguide core segment in layer II (dark grey) which is formed through a single added spin step of high refractive index PDMS (see Methods section).

The obvious Litmus test for photonic functionality of the lightvalve is operation as an on-off switch, which is reported in Fig. 2c for a 0.6 mm long valve. The top trace shows the temporal pressure sequence for the valve and the two bottom traces show the optical transmission across the valve in push-down (middle, red) and lift-up (bottom, blue) modes. Successful and repeatable switching with excellent extinction is observed for both pressure modes. Cycle rates can reach ~100 Hz and are limited by the microfluidic control system. The switches operated without degradation for over 100,000 switching cycles in both modes.

Next, we analyzed the on-off optical switching efficiency for different length lightvalves operated in lift-up mode. The results are displayed in Fig. 2d and show a steep increase in performance at around 500 μm length (with control height, *h*_*c*_ = 100 μm). This is due the fact that optical switching in lift-up operation relies on bending of the entire membrane formed by layer II; as such, when the effected membrane bend is small, optical rejection is low. Figure 2d shows that the lightvalve switches off for length/height (*L*_*v*_*/h*_*c*_) ratios above 5 and the on-off ratio continues to improve up to *L*_*v*_*/h*_*c*_~10. At even longer lengths, on-off ratios become inconsistent due to membrane deformations during actuation.

Push-down operation, on the other hand, is relatively length-independent as it relies only on deformation of the waveguide structure at the beginning of the lightvalve, which leads to poor mode coupling between the excitation and valve waveguides. Figure 2d shows that the on-off ratio depends on the applied pressure for a short valve length, *L*_*v*_ = 300 μm. After first reaching a maximum at 3psi due to optimized optical mode coupling, the transmission drops dramatically, resulting in an on-off ratio of ~45 dB at 40 psi, indicating excellent light blocking capability over short valve lengths length.

**Figure 3:**
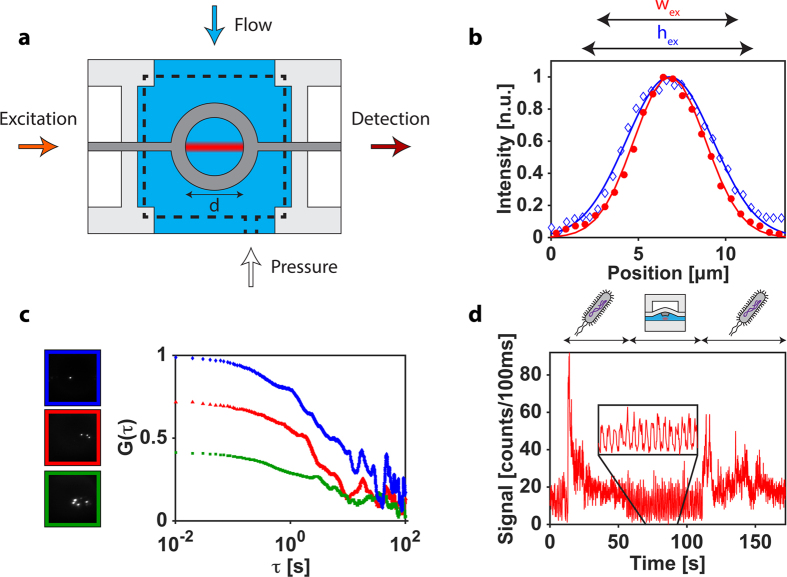
Lightvalve particle trap. (**a**) Top-down schematic of lightvalve trap with excitation laser light entering the trapping region from a solid-core waveguide, while signal is collected, filtered, and detected from the opposite waveguide facet. The observation volume is indicated in the center of the lightvalve trap by a Gaussian blurred red bar and is distinctly smaller than the trap volume which is characterized by the diameter (d) of the trap. Pressure is applied to the control layer, indicated here by dashed black lines. (**b**) Waveguide mode cross-sections, with the 1/e^2^ widths in the vertical (h_ex_ = 9.9 μm) and horizontal (w_ex_ = 8.2 μm) directions, define the optical interrogation region. (**c**) Autocorrelation curves for different numbers of particles trapped inside a lightvalve, d = 50 μm. Microscope images in the left are snapshots of fluorescent particles within the excitation volume for each autocorrelation trace. (**d**) Fluorescence signal of trapped, single *E. coli* bacteria. Illustrations above the fluorescence trace indicate when the trap is closed, containing a cell (bacterium cartoon), and when the trap is cycling (open lightvalve cartoon).

Finally, we demonstrate an implementation of the lightvalve as a functional element that unites both fluid handling and photonic functions of a biodetection assay. To this end, the lightvalve is built as an annular structure shown schematically in Fig. 3a. Fluidically, the lightvalves can be used to mechanically trap objects within the annulus when lowered into the channel. We fabricated annuli with 5–80 μm diameters, enclosing volumes between 140 fL and 35 pL. The lightvalves also act as peristaltic pumps for refreshing fluid within the traps by connecting three or more valves in series and actuating them sequentially in lift-up mode. Optically, the annulus enables in-plane optical interrogation of trapped particles using light that traverses the valve ring along the straight waveguide path. The optical path shown in Fig. 3a defines the optical excitation and collection volume of the trap. The solid-core waveguides are narrow enough to create effectively single vertical and lateral optical modes as shown in Fig. 3b. This allows for implementing advanced optical spectroscopy methods on small numbers of particles trapped inside the annulus. We illustrate this capability using fluorescence correlation spectroscopy (FCS). Figure 3c left shows top-down camera images of 3, 5, and 10 trapped, fluorescent microbeads (note that only beads within the excitation volume are fluorescing in the image). The corresponding FCS traces—acquired by in-plane fluorescence detection along the solid-core PDMS waveguides—are shown on the right. When the ratio of physical trap volume and optical excitation volume *V*_*exc*_ is taken into account, the particle concentration *c* obtained from the FCS curves (*c* *=* *G*(*0*)/*V*_*exc*_) agrees well with the value obtained by camera observation.

Lastly, we demonstrate the lightvalve trap’s ability to analyze single, trapped bioparticles – here, fluorescently stained *E. coli* bacteria. Figure 3d shows the time-dependent fluorescence signal collected from the trap. An initially empty trap is closed at t ~15 sec and a single *E. coli* bacterium is trapped within the observation volume. The detected fluorescence decreases continuously over the bacterium’s 40 second residence within the trap due to photobleaching. After 55 seconds, the bacterium is released by activating the lift-up function, followed by a series of actuations (i.e. fluid pumping) in search of another bacterium. The inset of Fig. 3d shows high signal when the trap is up, and low signal when trap is down. After 110 seconds, the trap is locked down again because a bacterium is detected above the background optical signal threshold. Subsequently, this bacterium is diffusing in and out of the observation volume, yielding a fluctuating fluorescence signal. We note that FCS analysis of the two bacteria trapped herein yield diffusion coefficients of ~0.5 μm^2^/s as expected for a particle of ~1 μm diameter.

## Discussion

In summary, we have introduced a new optofluidic platform that seamlessly marries optical and fluidic functions in a single chip. Based on combining solid- and liquid-core PDMS waveguides whose fabrication is compatible with purely microfluidic chips, we created devices that offer multi-modal photonic reconfigurability using core liquids, mechanical pressure and motion. The potential of this approach was illustrated using widely tunable liquid-core MMI waveguides and by the introduction of novel lightvalves that regulate both liquid and light flow. Extremely efficient optical switching and definition of physical particle traps for optical analysis were demonstrated. The fluidic valve shape and optical pathways created by the lightvalve can be designed independently and with great flexibility, making the lightvalve a powerful building block for future optofluidic devices.

## Methods

### Fabrication

**Figure 4:**
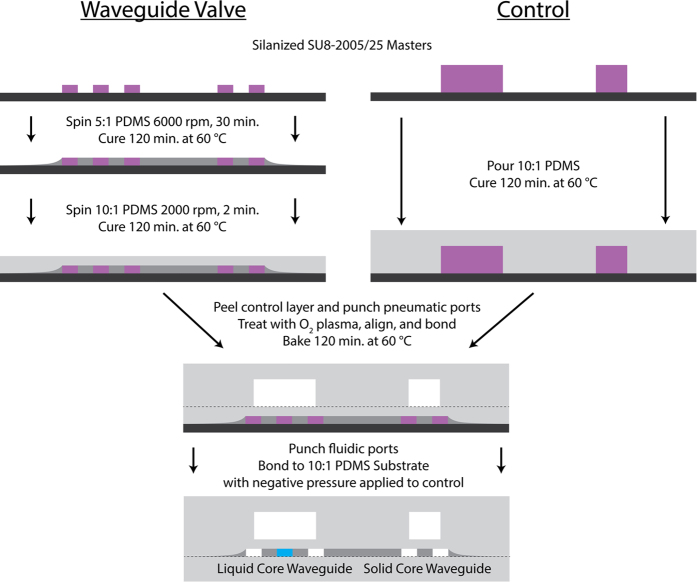
Fabrication of PDMS Waveguide Platforms. A stepwise fabrication workflow is presented, with high index PDMS colored dark grey, low index PDMS in light gray, SU8 in purple, and silicon in black. Dashed lines indicate bonding surfaces.

**Figure 5:**
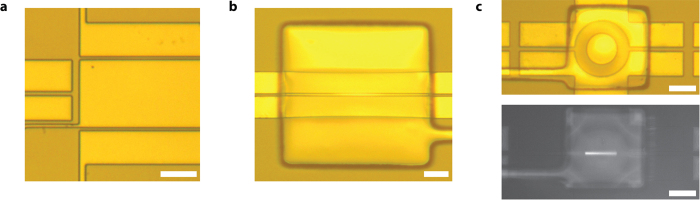
Example PDMS Waveguide Platform Microscope Images. **(a**) Reconfigurable optofluidic MMI, empty, (**b**) Lightvalve in its static state, empty, and (**c**) Lightvalve trap, both empty (top) and filled and under optical excitation (bottom). The filling fluid for the lightvalve trap is a 3 μM Dylight 488 solution and exhibits fluorescence when excited by 488 nm laser light, injected from the left solid-core waveguide. Note: all white scale bars represent 50 μm.

The optofluidic chips were fabricated using soft lithography. Seen in Fig. 4, the workflow involves parallel fabrication of waveguide valve and control layers. The solid-core optical waveguides are fabricated by spinning 5:1 (base:curing agent) PDMS (Sylgard) onto a 7 μm tall silanized^31^ SU-8 master (Microchem) at 6000 RPM for 30 minutes (spin speed and duration were optimized to minimize residual PDMS on top of SU-8 features). A 2-hour cure at 60 °C ensures full polymerization of the waveguide core material. A subsequent 2 minute, 2000 RPM spin of 10:1 PDMS then creates a continuous membrane across the waveguide valve layer. This layer structure preserves the optical waveguide properties as the polymer is transparent throughout the optical spectrum^32^ and 10:1 PDMS has a lower refractive index (n_10:1_≈1.420) than 5:1 PDMS (n_5:1_≈1.425)^25^. In parallel to waveguide fabrication, the control layer is fabricated by pouring and curing 10:1 PDMS on a silanized SU-8 master with 80 μm tall features. Once cured, the PDMS layer is peeled from the SU-8 master mold and ports (d = 1 mm) are punched to enable pneumatic access. After punching, the bottom of the control layer and top of the waveguide/fluidic layer are activated via oxygen plasma (30 sec at 60 W power), aligned on a custom alignment stage, and brought into contact, whereupon the bond is enhanced via a 2-hour thermal activation in a 60 °C oven. Next, ports are punched into the stack to allow fluidic access, followed by another peeling and bonding process. This step occurs with negative pressure applied to the pneumatic ports to prevent the bonding of waveguide valve layer to the substrate layer. In the case of single layer devices (i.e. tunable optofluidic MMI), only the left hand side of Fig. 4 is followed, replacing the 10:1 PDMS spin step with a drop casting of 10:1 PDMS. Waveguide chips were diced using commercial razor blades to ensure good facet quality for low optical coupling losses^33^. Microscope images of three completed devices are shown in Fig. 5.

### Experimental Setups

The optofluidic chips were stabilized by custom laser-cut acrylic manifolds, designed for simultaneous fluidic, pneumatic, and optical access. The chips were pneumatically operated using a custom control box (National Instruments and SMC), interfaced via Labview. All optical experiments used fiber-coupled laser excitation sources that were butt coupled to the PDMS optofluidic devices at the solid-core waveguide facets. Fiber vibrations were remediated by touching the fiber facet to the waveguide facet. In-plane signal was collected via objective (Newport) at the waveguide facet, spectrally filtered (filters varied depending on excitation/emission, Semrock), and focused into a connectorized multimode fiber that was attached to a single photon avalanche photodiode (Excelitas). A time correlated, single photon counting card—operated in time-tagged time-resolved mode—was used to accumulate and store the signal for downstream processing (Picoquant). Out-of-plane chip monitoring and signal collection was simultaneously achieved using a custom compound microscope^34^.

Finite difference method (FDM) optical simulations of the liquid-core MMI waveguides were performed using Fimmwave, a commercial photonic design software (Photon Design). DH5α *E. coli* staining was accomplished using 50 μM Syto62 (Invitrogen). Once the nucleic acid was stained, the bacteria were pelleted, the excess dye was removed and replaced with T50 buffer, and the bacteria were injected into the fluidic inlet of the lightvalve trap device.

## Additional Information

**How to cite this article**: Parks, J. W. and Schmidt, H. Flexible optofluidic waveguide platform with multi-dimensional reconfigurability. *Sci. Rep*. **6**, 33008; doi: 10.1038/srep33008 (2016).
